# A dehydrin-dehydrin interaction: the case of SK_3_ from *Opuntia streptacantha*

**DOI:** 10.3389/fpls.2014.00520

**Published:** 2014-10-10

**Authors:** Itzell E. Hernández-Sánchez, David M. Martynowicz, Aida A. Rodríguez-Hernández, Maria B. Pérez-Morales, Steffen P. Graether, Juan F. Jiménez-Bremont

**Affiliations:** ^1^Laboratorio de Estudios Moleculares de Respuesta a Estrés en Plantas, División de Biología Molecular, Instituto Potosino de Investigación Científica y Tecnológica ACTangamanga, México; ^2^Department of Molecular and Cellular Biology, University of GuelphGuelph, ON, Canada

**Keywords:** yeast two-hybrid, SK_3_-dehydrin, K-segments, homodimer, histidine-rich region, intrinsically disordered proteins

## Abstract

Dehydrins belongs to a large group of highly hydrophilic proteins known as Late Embryogenesis Abundant (LEA) proteins. It is well known that dehydrins are intrinsically disordered plant proteins that accumulate during the late stages of embryogenesis and in response to abiotic stresses; however, the molecular mechanisms by which their functions are carried out are still unclear. We have previously reported that transgenic Arabidopsis plants overexpressing an *Opuntia streptacantha* SK_3_ dehydrin (OpsDHN1) show enhanced tolerance to freezing stress. Herein, we show using a split-ubiquitin yeast two-hybrid system that OpsDHN1 dimerizes. We found that the deletion of regions containing K-segments and the histidine-rich region in the OpsDHN1 protein affects dimer formation. Not surprisingly, *in silico* protein sequence analysis suggests that OpsDHN1 is an intrinsically disordered protein, an observation that was confirmed by circular dichroism and gel filtration of the recombinantly expressed protein. The addition of zinc triggered the association of recombinantly expressed OpsDHN1 protein, likely through its histidine-rich motif. These data brings new insights about the molecular mechanism of the OpsDHN1 SK_3_-dehydrin.

## Introduction

Plants have developed a wide variety of mechanisms to cope with environmental stresses; one response to these adverse conditions is the activation of a group of stress response genes, including Late Embryogenesis Abundant (LEAs) proteins. LEA group II proteins, also known as dehydrins (DHNs), are a plant protein family that is typically induced in response to stress conditions that cause cellular dehydration, such as low temperatures, high salinity and drought (Battaglia et al., 2008; Reyes et al., 2008). All members of the DHN family possess at least one amphipathic α-helix forming domain named the K-segment (EKKGIMDKIKEKLPG), which can interact with macromolecules and specific membrane regions to protect them against damage (Koag et al., 2009). Other conserved motifs such as the S- and Y-segments, and the ϕ-segment, have also been described in this group of proteins. Phosphorylation of the S-segment is associated with its transport to the nucleus and/or cation binding; the Y-segments display similarity to the nucleotide-binding site present in chaperones (Sun and Lin, 2010). The ϕ-segments are poorly conserved in sequence and length; however these segments are important to maintain the unstructured state and the cryoprotective activities of DHNs (Close, 1996, 1997; Hughes and Graether, 2011).

Other segments in DHNs sequence have been described but are likely not as common as the Y-, S-, and K-segments. One example is the histidine-rich regions (H-X3-H, HH, and H_n_), which allow for the formation of complexes with metal ions (Hara et al., 2001, 2005; Hara, 2010). Also, lysine-rich sequences are found in many types of DHNs. This segment is known as Poly-K or KEKE motif (KKKKKKEKKK), which may function as a polar zipper that interacts with polar KEKE motif present in other proteins or with DNA (Hara et al., 2009). The detection of these new segments suggests novel functions for DHNs, as chelators and DNA binding proteins.

Evidence from both experimental and bioinformatics analyses show that DHNs are intrinsically disordered proteins (IDPs) or mostly unstructured with a minor inclusion (0.7–5%) of α-helices (Tompa et al., 2006). This unstructured state provides functional benefits, such as: an increased interaction surface area, conformational flexibility to interact with several targets, the presence of molecular recognition elements that fold upon binding and accessible post-translational modification sites (Gsponer et al., 2008). These unstructured states may facilitate the protective functions of DHN proteins and their remarkable functional versatility.

Despite the diverse functions proposed for DHNs, the mechanisms by which they enhance stress tolerance have not yet been fully elucidated. Several biological processes such as signal transduction, transcription, and stress responses are mediated by physical interactions between proteins. We have previously reported that transgenic Arabidopsis plants overexpressing *Opuntia streptacantha* SK_3_ DHN (OpsDHN1) show enhanced tolerance to freezing stress (Ochoa-Alfaro et al., 2012).

Here, we characterize the protein dimerization of OpsDHN1 using a split-ubiquitin yeast two-hybrid system, and also by size-exclusion chromatography of the recombinant protein. In this regard, we find that deletion of regions that contain the conserved motifs present in OpsDHN1 protein affects dimer formation. In addition, the intrinsically disordered state of the recombinantly expressed protein is analyzed by circular dichroism (CD) and gel filtration. These data bring new insights about the molecular mechanism of OpsDHN1 SK3-dehydrin, opening a new hypothesis about the function of this protein.

## Materials and methods

### Yeast strains and medium

All yeast strains were generated from *Saccharomyces cerevisiae* NMY51 parental strain, and are listed in Supplemental Table 1. Transformed yeast cells were selected on synthetic dextrose medium [SD, containing yeast nitrogen base without amino acids (YNB, 6.7 g/L, dropout mix 0.6 g/L and, glucose 20 g/L]. The amino acids and nucleotides: leucine 0.1 g/L, tryptophan 0.02 g/L, histidine 0.02 g/L (LWH), uracil 2 mg/L (U) and adenine 0.01 g/L (A) were supplemented to the SD medium to provide the appropriate auxotrophic components. For solid plates, 2% agar was added to the medium.

### Split-ubiquitin constructs

The open reading frame of OpsDHN1 (accession no. HO058650) (747 bp) was amplified from entry plasmid pCR8-OpsDHN1 (Ochoa-Alfaro et al., 2012) by PCR using Phusion High-fidelity DNA polymerase (Thermo scientific®). The PCR product was digested with *Sfi*I enzyme, and generated fragment was cloned in-frame into their respective DUALhunter vectors pDHB1-Cub and pPR3-N-NubG. To determinate which regions are implicated in the OpsDHN1-OpsDHN1 protein interaction, three constructs were derived from the OpsDHN1 ORF. The version OpsDHN1-SK_2_ (residues 1–199), OpsDHN1-S (residues 1–97) and OpsDHN1-S(ΔH)K_3_ (residues 1–111/138–248) constructs were amplified by high fidelity PCR. The OpsDHN1-S(ΔH)K_3_ version was generated by PCR using the bases 1–333 and 415–747 from the OpsDHN1 ORF. In order to fuse both DNA fragments, *Kpn*I restriction sites were included in the primers sequences. After amplification, both PCR products were digested with *Kpn*I enzyme (Invitrogen) to generate cohesive ends; both digested fragments were fused using T4 DNA ligase (Invitrogen). All OpsDHN1 constructs were directionally cloned into the respective DUAL hunter (pDHB1-Cub and pPR3-N-NubG) vectors using *SfiI* restriction sites. The primers sequences are listed in Supplemental Table 2. All constructs were confirmed by DNA sequencing.

### Yeast two-hybrid analysis

Protein interactions between OpsDHN1 and OpsDHN1 constructs were examined in yeast using the DUALhunter kit (Dualsystems Biotech), according to the manufacturer's protocol. To confirm correct expression and functionality of the system, each of the bait constructs [OpsDHN1 SK_3_-Cub, S(ΔH)K_3_-Cub, SK_2_-Cub and S-Cub] were co-transformed with prey control vectors, which express a fusion of endogenous ER protein Alg5 to the wild-type NubI (positive) portion or to the NubG (negative) portion bearing the isoleucine to glycine mutation. The OpsDHN1 SK_3_-Cub construct was co-transformed with pPR3-N and growth on SD-LWHA medium supplemented with different amounts (25, 35, 45, 55, and 65 mM) of 3-aminotriazole (3-AT, a competitive inhibitor of the *HIS3* gene product) to determine the concentration required for testing the interaction OpsDHN1-OpsDHN1. In the DUALhunter system, protein interaction leads to an activation of the *lacZ*, *HIS3*, and *ADE2* reporter genes. In this sense, each bait construct was co-transformed with each prey construct in the *Saccharomyces cerevisiae* NMY51 strain, plated on SD medium without Leu and Trp (SD-LW, to select for transformed yeast), and grown at 28°C for 4 d. For the screen interaction, transformed yeast cells were grown on SD liquid medium without LWHA to an OD600 of 0.8. Five microliters of different dilutions (1:10, 1:100, and 1:1000) were spotted on solid SD-LWHA medium supplemented with 45 and 55 mM 3-AT plates, and grown for 5 days at 28°C. As a positive interaction control we co-transformed yeast cells with SV40 LargeT antigen (Cub) and tumor suppressor Δp53 (NubG) vectors (Dualsystems Biotech).

### β-galactosidase activity assay

The activity of the β-galactosidase enzyme activity was analyzed qualitatively by the X-gal assay (Möckli and Auerbach, 2004). Yeast strains expressing the different versions of the OpsDHN1 protein (Supplemental Table 1) were selected and inoculated into 5 mL of SD-LWHA liquid medium supplemented with 45 and 55 mM 3AT. The yeast cells were grown overnight until they reached an OD600 of 0.8. Subsequently, 1 mL of each culture was centrifuged at 3000 rpm for 5 min. The supernatant was decanted and we carried out cell lysis by two cycles of freeze-thaw (3 min in liquid nitrogen and then 3 min at 37°C). Finally, the pellets were resuspended in 20 μL of distilled water, transferred to a 96 well plate and mixed with 100 μL of PBS buffer pH 7.4 containing: 500 μg/mL of X-gal, 0.5% (w/v) agarose and 0.05% (v/v) β-mercaptoethanol. The samples were incubated at room temperature and the enzyme activity was monitored for blue color development after 30 min.

A quantitative *o*-Nitrophenyl β-galactoside (ONPG) assay was conducted, 0.01 mL of yeast transformants extracts cells was added to final volumen of 0.8 mL of Z buffer (60 mM Na2HPO4, 40 mM NaH2PO4, 10 mM KCl, 1 mM MgSO4, and 38.3 mM 2-mercaptoethanol, pH 7). All reactions were initiated upon addition of 0.2 mL of ONPG substrate (4 mg/mL) and concluded with 0.4 mL of 1M Na_2_CO_3_. β-Galactosidase activity was calculated using the formula (1.7^*^OD420)/(0.0045^*^volume^*^[protein]^*^time) with volume of extract added in mL, [protein] in the extract in mg/mL, and time of reaction in min (nmol ONP/min per mg of protein). Reactions were terminated within the linear range of the assay (OD420 < 1.0).

### Cloning, expression, and purification of recombinant OpsDHN1 and other proteins

The OpsDHN1 was subcloned from the plasmid pCR8 containing the gene and inserted into the pET-22b plasmid between the *NdeI* and *HindIII* cut sites using standard molecular biological methods. Briefly, the OpsDHN1 gene was amplified by PCR using the forward primer 5′-GTAGCATATGGCGGAAGAACACCAAAAAGGTG-3′ and the reverse primer 5′-CAGCAAGCTTTTAAGTTGATGAAGGGGGTTGATCACAC-3′ (restriction sites are underlined) using PfuTurbo (Stratagene, La Jolla, CA) according to the manufacturer's recommended protocol. The PCR product was digested with *NdeI* and *HindIII* restriction enzymes and ligated into the pET-22b expression vector (Novagen, Gibbstown, NJ) that had also been digested with *NdeI* and *HindIII*.

The pET-22b-OpsDHN1 construct was transformed into *E. coli* BL21 (DE) cells and grown overnight in 5 mL Lisogeny broth (LB, 10 g/L peptone, 5 g/L yeast extract, 10 g/L NaCl (and 15 g/L agar for solid medium) containing 50 μg/mL ampicillin. The next morning, 2 × 0.5 mL of the overnight culture was transferred to 2 × 500 mL of LB containing 50 μg/mL ampicillin. The large-scale culture was shaken at 250 rpm at 37°C until on OD600 of ~0.8 was reached, at which point isopropyl β-D-1-thiogalactopyranoside (IPTG) was added to a final concentration of 0.4 mM. After continuing growth for an additional 3 h, the bacterial cells were centrifuged at 6000x*g* for 15 min. Pellets were stored overnight at −20°C.

Pellets were resuspened in 15 mL milli-Q grade water and one protease inhibitor tablet with EDTA (Roche Diagnostics) was added. The suspension was boiled for 20 min to lyse the cells and denature most cellular proteins before being cooled at −20°C for 10 min (Livernois et al., 2009). Tris-HCl (pH 8.0) and imidazole were added to give final concentrations of 20 and 10 mM, respectively. To remove insoluble membranes and denatured proteins, the sample was centrifuged at 70,000×*g* for 30 min at 4°C. To further remove insoluble material, the sample was passed through a 0.22 μM syringe filter.

Given the high number of histidine residues in the protein sequence, OpsDHN1 was purified by passing the sample through a 2 mL GE Healthcare HisTrap column (Baie d'Urfe, QC, Canada). After washing the column with 10 volumes of buffer A (20 mM Tris pH 8.0, 500 mM NaCl, 10 mM imidazole), the protein was eluted using a linear gradient from buffer A to buffer B (buffer A with 500 mM imidazole) over 5 column volumes. Fractions were run on a SDS polyacrylamide gel and samples containing protein were pooled and lyophilized. The OpsDHN1 protein was desalted and further purified by using reversed-phase HPLC as was performed for the K_2_ dehydrin (Hughes and Graether, 2011). Fractions were run on a SDS polyacrylamide gel and those fractions containing pure OpsDHN1 were pooled, lyophilized, and stored at −20°C until further use.

*Vitis riparia* K_2_ protein was purified as previously described (Findlater and Graether, 2009). The cloning of the *Drosophila melanogaster* FROST protein will be described elsewhere (Graether et al., unpublished results).

### Biochemical characterization of OpsDHN1

Circular dichroism data for OpsDHN1 was collected using a Jasco-815 CD spectropolarimeter (Easton, MD). The protein was dissolved in 10 mM sodium phosphate, pH 7.4 at a concentration of 5.5 μM. A quartz cuvette with a 2 mm pathlength (Hellma, Concord, ON, Canada) containing the OpsDHN1 sample was scanned from 250 to 190 nm. Scans were repeated in the presence of 1 mM ZnCl_2_, 50 mM SDS, and 1 mM ZnCl_2_/50 mM SDS. The spectra were averaged over eight accumulations.

Samples containing 200 μg OpsDHN1 alone or in the presence of 1 mM ZnCl_2_ were injected onto a Superdex G75 gel filtration column. The samples were eluted at a flow rate of 0.8 mL/min using a 50 mM sodium phosphate, pH 7.4, 150 mM NaCl buffer. The column was calibrated with a series of protein standards using the same buffer (i.e., with and without ZnCl_2_) to obtain the relationship between the elution volume and Stokes radius. The hydrodynamic radii of lysozyme (Parmar and Muschol, 2009), carbonic anhydrase (Potschka, 1987), ovalbumin (Andrews, 1970), BSA (Andrews, 1970), β-casein (Marchesseau et al., 2002), and glycogen phosphorylase (DeVincenzi and Hedrick, 1967) were taken from the literature.

The cryoprotection assay was performed as described previously (Hughes and Graether, 2011). For the assay, LDH was prepared by overnight dialysis against 10 mM sodium phosphate buffer, pH 7.4. The dehydrins and reagents were dissolved in 10 mM sodium phosphate, pH 7.4 buffer. LDH samples (7.5 μL of 50 μg/mL) were mixed with the OpsDHN1, *Vitis riparia* K_2_, or FROST protein (7.5 μL) or buffer only (7.5 μL) in a 1.5 mL microcentrifuge tube. The concentration of LDH in all of the freeze-thaw assays was 0.18 μM. The samples were immersed five times in liquid nitrogen for 30 s and thawed by immersion in a circulating water bath at 4°C for 5 min. LDH activity was measured by diluting the enzyme to 0.5 μg/mL into 750 μL of 10 mM sodium phosphate, pH 7.4, 2 mM NADH, 10 mM pyruvic acid. Enzyme activity was followed on a Cary 100 spectrophotometer (Varian, Mississauga, ON, Canada) by measuring absorbance at 340 nm to follow the disappearance of NADH. Data from the assays are plotted as percent recovery of LDH activity vs. additive concentration. The results were fitted to:

$$\%\text{LDH}\;\text{recovery}=\frac{a}{1+e^{\frac{-\;(x-x_{0})}{b}}}$$

where *x* is the additive concentration, *x*_0_ is the percent recovery in the absence of the additive, and *a* and *b* are fitted variables. The results were fitted to Hughes and Graether (2011).

## Results

### Dehydrin-dehydrin interaction: OpsDHN1

**Figure 1:**
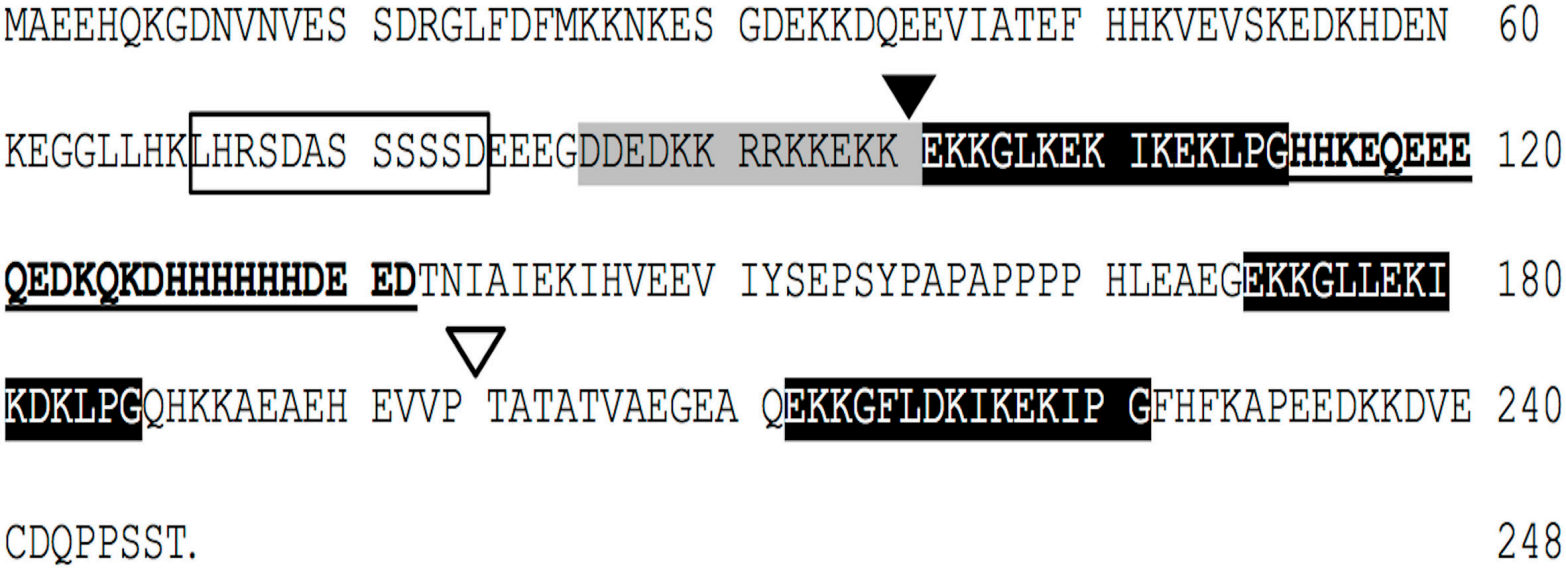
**The *Opuntia streptacantha* DHN (OpsDHN1) amino acid sequence, and schematic representation of characteristic motifs present in OpsDHN1 protein: S-segment (open box), poly-lysine rich sequence (gray boxes), K-segments (black boxes), and histidine-rich motif (in bold)**. The end of S version (black triangle), and the end of SK_2_ version (open triangle); histidine rich region deleted (underlined sequence).

We have previously reported on an *O. streptacantha* dehydrin gene, encoding an SK_3_-type acidic protein (Ochoa-Alfaro et al., 2012; Jiménez-Bremont et al., 2013). This OpsDHN1 protein contains three K-segments (lysine-rich repeat) at the central (residues 98–112) and C-terminal regions (residues 172–186 and 212–226), and a serine repeat segment (S-segment) located at residues 75–79. A poly-lysine rich sequence (Poly-K), also known as the KEKE motif (DDEDKKRRKKEKK), is located between the S-segment and the first K-segment (residues 85–100). A histidine-rich motif (KQKDHHHHHHDEED), characteristic of metal binding proteins, was identified between the first and second K-segment (residues 112–137; Figure 1).

In order to provide new aspects about the dehydrins, we explored a possible homodimerization state of the OpsDHN1. We employed the split-ubiquitin yeast two-hybrid system (Dualsystems biotech; Supplemental Figure 1) to avoid auto-activation for acidic proteins reported in the classic GAL4 yeast two-hybrid systems (Möckli et al., 2008).

**Figure 2:**
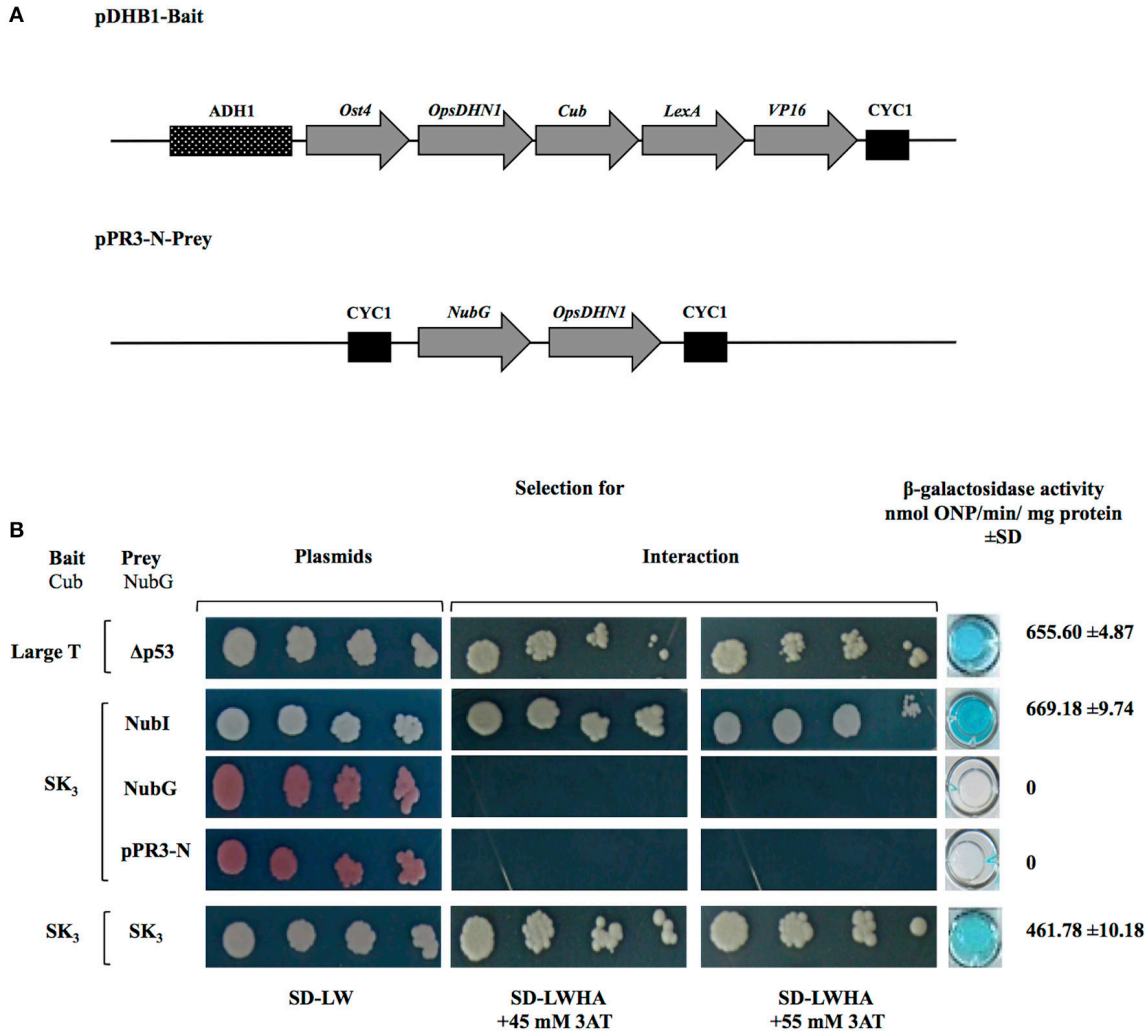
**OpsDHN1-OpsDHN1 protein interaction using the split-ubiquitin yeast two-hybrid assay**. **(A)** Schematic representation of OpsDHN1 bait and prey constructs. In the pDHB1 construct, the *OpsDHN1* gene was fused at its N-terminal to the Ost4 membrane protein, and its C-terminal to the ubiquitin C-terminal moiety. LexA-VP16, the artificial transcription factor, is fused at the C-terminal end of ubiquitin. This construct is under control of the ADH1 promoter region and CYC1 terminator region. In the pPR3-N-prey construct, the ubiquitin N-terminal was fused to the *OpsDHN1* gene. This construct is under control of CYC1 promoter region and CYC1 terminator region. **(B)** Yeast cells carrying the control system interaction LargeT-Cub/Δp53-NubG, and functional assay of OpsDHN1 SK_3_ as bait construct using prey control system vectors: NubI, NubG and pPR3-N, and OpsDHN1-Cub/OpsDHN1-NubG interaction. Yeast strains was plated to an OD600 of 0.8, and at serial 10-fold dilutions on semi-selective (SD-LW) and on selective (SD-LWHA) media supplemented with 3-AT (45 and 55 mM). Quantitative β-Galactosidase activity was assayed by hydrolysis of the o-nitrophenyl-b-galactoside (ONPG), as expressed in nmol ONP/min per mg of protein. Data represent the mean ± *SD*, (*n* = 3).

The open reading frame of *OpsDHN1* gene was cloned in both bait and prey vectors (Figure 2A). As a positive control, the interaction between LargeT antigen (Cub) and Δp53 (NubG) was included. When we analyzed the interaction between OpsDHN1 SK_3_-Cub bait and OpsDHN1 SK_3_-NubG prey, homodimer formation was detected (Figure 2B). The interaction strength of dimer (OpsDHN1-OpsDHN1) was comparable to the positive control of interaction, in the growth on selective medium SD-LWHA supplemented with concentrations of 3AT (45 and 55mM). Respect to β-galactosidase activity, the OpsDHN1-OpsDHN1 interaction showed 461.78 ± 10.18 in comparison with 655.60 ± 4.87 nmol ONP/min/mg protein correspond to LargeT-Cub/Δp53-NubG interaction (Figure 2B). Co-expression of OpsDHN1 SK_3_-Cub construct with the control positive vector (NubI) resulted in growth on SD-LWHA medium and color development in a β-galactosidase assay. When SK_3_-Cub bait construct was co-transformed with the negative control (NubG and pPR3-N) vectors no growth or color development was observed (Figure 2B; Supplemental Figure 2A). These results suggest that OpsDHN1 interacts with itself in yeast.

### Regions involved in OpsDHN1-OpsDHN1 protein interaction

**Figure 3:**
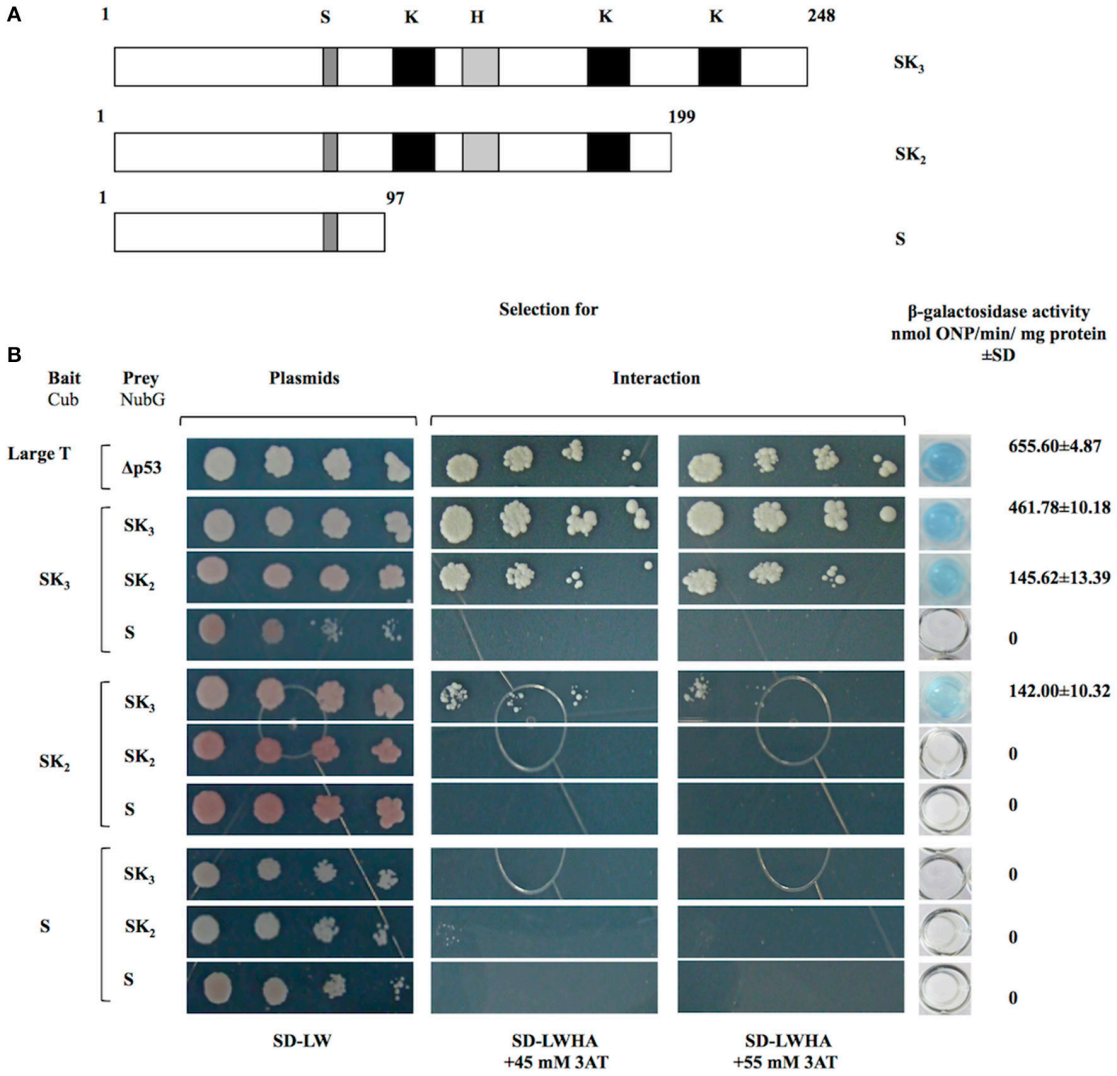
**Interaction analyses among OpsDHN1 (SK_3_) and truncated versions (SK_2_ and S) using the split-ubiquitin yeast two-hybrid assay**. **(A)** Schematic representations of OpsDHN1 derived versions used in the two-hybrid split ubiquitin system. OpsDHN1 SK_3_ full version; OpsDHN1 SK_2_ C-terminal truncated version (residues 1–199); OpsDHN1 S short version (residues 1–97). The capital letters indicates characteristic motifs of OpsDHN1 protein: S (S-segment), K (K-segment), and H (histidine-rich motif). **(B)** Yeast cells carrying the control system interaction LargeT-Cub/Δp53-NubG. Interaction analysis among baits and preys OpsDHN1 derived versions (SK3, SK2, and S). Yeast strains was plated to an OD600 of 0.8, and at serial 10-fold dilutions on semi-selective (SD-LW) and on selective (SD-LWHA) media supplemented with 3-AT (45 and 55 mM). Quantitative β-Galactosidase activity was assayed by hydrolysis of the *o*-nitrophenyl-b-galactoside (ONPG), as expressed in nmol ONP/min per mg of protein. Data represent the mean ± *SD*, (*n* = 3).

To determine whether K-segments are involved in OpsDHN1-OpsDHN1 protein interaction, we designed two truncated versions derived from full length OpsDHN1-SK_3_ protein. OpsDHN1-SK_2_ version was constructed by removing the C-terminal region (residues 200–248); the deleted region includes the last K-segment (Figure 3A). In the OpsDHN1-S version we deleted residues 98–248 containing three K-segments and histidine-rich motif, keeping only the S-segment and KEKE motif (Figure 3A). These versions were cloned in both prey and bait vectors, and assayed in the split-ubiquitin system. The correct expression and no auto-activation of reporter genes by SK_2_-Cub and S-Cub bait constructs were evaluated by the control assay (Supplementary Figures 2B,C).

The removal of C-terminal region that include the last K-segment (SK_2_) resulted in a decrease of yeast growth co-transformed with SK_3_-Cub/SK_2_-NubG on SD-LWHA/3-AT medium, and also a reduction in the enzyme activity, where the interaction SK_3_-Cub/SK_2_-NubG or SK_2_-Cub/SK_3_-NubG showed 145.62 ± 10.32 and 142.00 ± 13.39 nmol ONP/min/mg protein, respectively, while the SK_3_-Cub/SK_3_-NubG full-length interaction presented 461.78 ± 10.18 and nmolONP/min/mg protein (Figure 3B). However, when the S-NubG version was co-expressed with SK_3_-Cub, no interaction was seen (Figure 3B). In addition, yeast co-expressing truncated versions: SK_2_-Cub/SK_2_-NubG, SK_2_-Cub/S-NubG, and S-Cub/S-NubG, were unable to grow on SD-LWHA/3-AT medium (Figure 3B). In order to confirm dehydrin interactions, we swapped all versions in the respective bait and prey vectors, and achieved similar results (Figure 3B). These findings suggest that region that comprises residues 98–248, which contains three K-segments and the histidine-rich motif, is involved in the OpsDHN1-OpsDHN1 protein interaction.

### The histidine-rich motif is important in OpsDHN1-OpsDHN1 protein interaction

**Figure 4:**
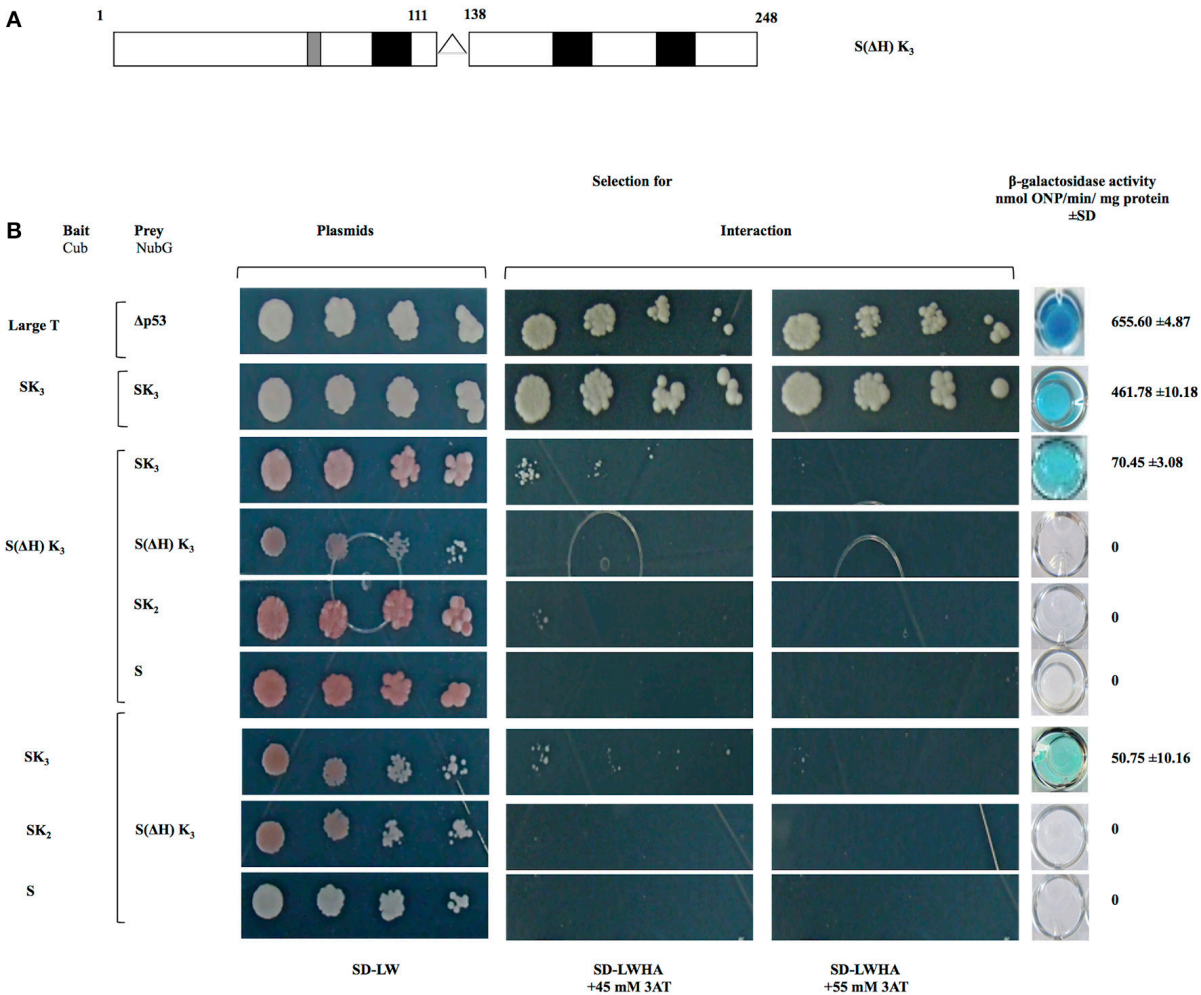
**Interaction analyses among S(ΔH) K_3_, OpsDHN1 (SK_3_), and truncated versions (SK_2_ and S) using the split-ubiquitin yeast two-hybrid assay**. **(A)** Schematic representations of the OpsDHN1 S(ΔH)SK_3_ version without histidine-rich motif (residues 1–111:128–248). **(B)** Yeast cells expressing the control system interaction LargeT-Cub/Δp53-NubG, OpsDHN1 SK_3_ full-length interaction. Interaction analysis among bait OpsDHN1 S(ΔH) K_3_-Cub with OpsDHN1-NubG [SK3, S(ΔH) K_3_, SK2, and S] prey vectors, interaction analysis of the swapped versions in the respective bait and prey vectors. Yeast strains was plated to an OD600 of 0.8, and at serial 10-fold dilutions on semi-selective (SD-LW) and on selective (SD-LWHA) media supplemented with 3-AT (45 and 55 mM). Quantitative β-Galactosidase activity was assayed by hydrolysis of the *o*-nitrophenyl-b-galactoside (ONPG), as expressed in nmol ONP/min per mg of protein. Data represent the mean ± *SD*, (*n* = 3).

To investigate the role of histidine-rich motif in the OpsDHN1-OpsDHN1 protein interaction, we generated a S(ΔH)K_3_ construct where the histidine-rich motif (residues 112–137) was deleted (Figure 4A). This version was cloned into bait and prey vectors and assayed in the yeast two-hybrid system. Correct expression and no auto-activation of reporter genes by OpsDHN1 S(ΔH)K_3_-Cub bait construct were evaluated in the control assay (Supplementary Figure 2D). We analyzed whether the deletion of histidine rich region in one copy of OpsDHN1 affected its interaction with the full length version. Co-expression of S(ΔH)K_3_-Cub/SK_3_-NubG constructs showed a severe decrease in yeast growth on SD-LWHA/3AT medium, and a reduction on β-galactosidase activity (70.45 ± 3.08 nmol ONP/min/mg protein; Figure 4), as a result of a reduction in the DHN-DHN interaction. Nevertheless, when we co-expressed S(ΔH)K_3_-Cub/S(ΔH)K_3_-NubG constructs, no interaction was detected. Furthermore, as observed in Figure 4B, no interaction was found when we used S(ΔH)K_3_-Cub with the truncated constructs (SK_2_-NubG or S-NubG)Similar behavior was obtained when we swapped all constructs in the respective DUAL hunter vectors (Figure 4B). These results suggest that the histidine-rich region play a key role in OpsDHN1 dimer formation, with other regions possibly providing minor contributions.

### Structure and function of recombinant OpsDHN1

Bacterial recombinant OpsDHN1 was first characterized by running the protein through a size-exclusion column. All previously characterized dehydrins are intrinsically disordered proteins (IDPs), and as such elute as much larger proteins than their sequences would predict. This is also the result we obtained for OpsDHN1 (Table 1). Based on the protein sequence, OpsDHN1 is predicted to have a molecular weight of 28.3 kDa, but the size-exclusion experiments shows that it has an apparent molecular weight of 135 kDa and a Stokes radius of 46.9 Å. The addition of zinc (1 mM) caused the profile of the protein to change dramatically. The narrow peak suggested that OpsDHN1 had compacted somewhat, resulting in an apparent molecular weight of 65.2 kDa. However, there is also a second, extremely broad peak centered around 1000 kDa, suggesting that the protein is highly associated in the presence of the metal ion.

**Table 1:** **Apparent molecular weight and Stokes radius of OpsDHN1 as determined by size-exclusion chromatography**.

**Condition**	**MW_**app**_ (kDa)**	**Stokes radius (Å)**
OpsDHN1	135	46.9
OpsDHN1 + 1 mM ZnCl_2_	65.2^*^	37.0

* A second, very broad peak was observed at 1000 kDa.

**Figure 5:**
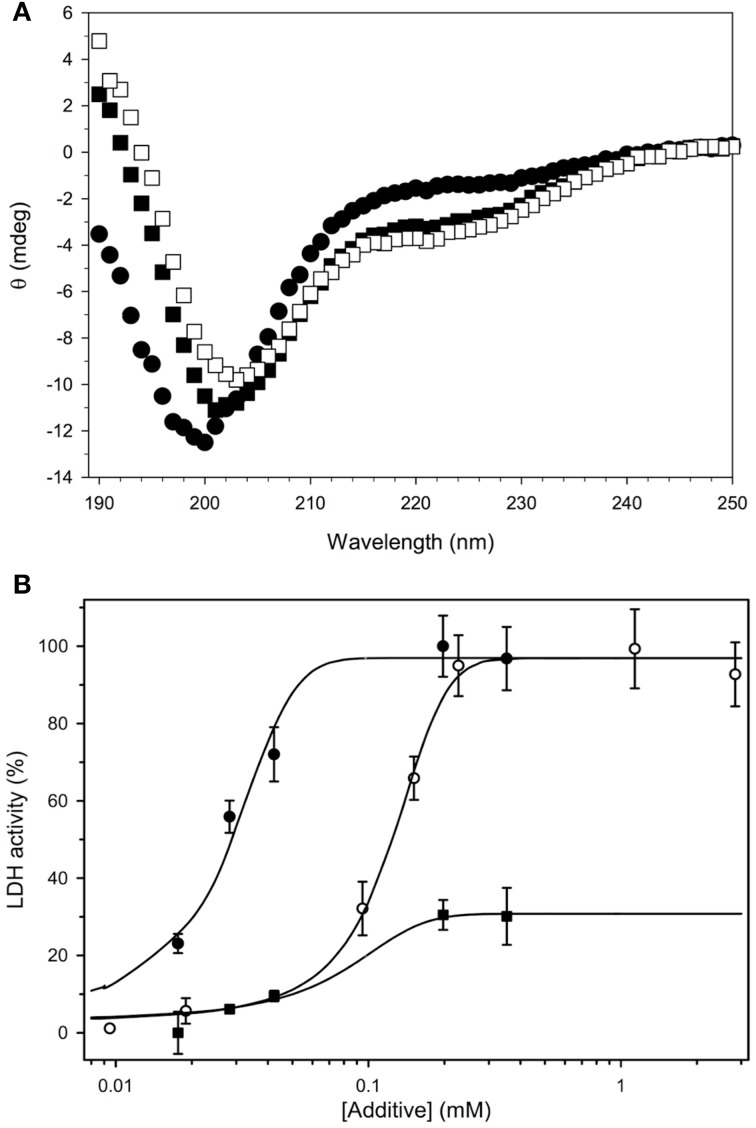
**Biochemical characterization of recombinant OpsDHN1**. **(A)** The secondary structure content of OpsDHN1 was determined under several conditions. OpsDHN1 alone, filled circles; OpsDHN1 in the presence of 50 mM SDS, filled squares; OpsDHN1 in the presence of 50 mM SDS and 1 mM ZnCl_2_, open squares. **(B)** Cryoprotection assay with OpsDHN1. The protection of LDH from freeze-thaw damage was assessed as described in the Materials and Methods. OpsDHN1, filled circles; *Vitis riparia* K_2_, open circles; FROST protein, filled squares.

Circular Dichroism (CD) was also used to support the idea that this dehydrin is also an IDP. For OpsDHN1, the protein in buffer alone shows a large minimal peak at 200 nm and very little ellipticity at 222 nm, demonstrating the protein contains a considerable amount of coil structure and very little ordered secondary structure such as an α-helix (Figure 5A, filled circles). The large number of histidine residues (19/248 residues; Supplemental Figure 3) suggested that this protein may bind metals, a property which was exploited in the purification protocol. In the presence of 1 mM ZnCl_2_, the CD spectra showed very little signal, possibly due to protein self-association (data not shown). Other studies have shown that some dehydrins are located close to membranes, and that the addition of the membrane-like SDS micelles causes dehydrin to gain helical structure (Koag et al., 2003). To examine whether this held true for OpsDHN1, we performed the CD experiments in the presence of 50 mM SDS (Figure 5A, filled squares). The loss of intensity of the minimum peak, its shift to 202 nm, and the increased signal at 222 nm suggest that OpsDHN1 is gaining some α-helical secondary structure when bound to a membrane. Interestingly, the simultaneous addition of SDS and zinc did not cause the protein to aggregate, but instead show that OpsDHN1 has still gained some α-helical structure (Figure 5A, open squares).

Other studies have shown that dehydrins are able to protect lactate dehydrogenase (LDH) from freeze-thaw damage. Previously, we have shown that the efficiency of protection depends on the size of the dehydrin (Hughes et al., 2013). Shown in Figure 5B is the cryoprotection assay results for OpsDHN1, the positive control protein K_2_, and the negative control protein FROST. The data show that OpsDHN1 is highly effective at preventing the denaturation of LDH from freeze/thaw damage. To recover 50% of lost enzyme activity, OpsDHN1 requires ~1/5 of the protein compared to *Vitis riparia* K_2_ dehydrin. FROST is an intrinsically disordered protein found in *Drosophila*, and like OpsDHN1 and K_2_, it is also a disordered protein. However, the data show that it is very ineffective at cryoprotection.

## Discussion

Dehydrins (DHNs) are a versatile protein group with multiple functions proposed from *in vitro* experiments, such as being cryoprotectants, chaperones, antioxidants, and ion sequestrants (Hara, 2010). DHNs accumulate to high amounts in all vegetative tissues under stress factors that cause cellular dehydration, such as drought, cold and high salinity (Allagulova et al., 2003; Kovacs et al., 2008). Transgenic plants that overexpress DHNs genes show an increased plant tolerance to abiotic stress (Xu et al., 1996; Hara et al., 2003; Yin et al., 2006; Peng et al., 2008; Ochoa-Alfaro et al., 2012). Despite progress toward the understanding the role of DHNs in stress tolerance, the molecular mechanisms by which these proteins exert their functions inside the cells remain unknown (Hanin et al., 2011).

We previously reported that the *O. streptacantha* SK_3_ DHN (OpsDHN1) is induced by cold, and plays a role in plant freezing stress tolerance (Ochoa-Alfaro et al., 2012; Jiménez-Bremont et al., 2013). In this study, we demonstrate that OpsDHN1 interacts with itself using a split-ubiquitin yeast two-hybrid system (Figure 2). Interestingly, the formation of dimers between dehydrins has also been reported in other plants. Rahman et al. (2013) demonstrated that the TsDHN-2 Y_2_SK_2_ type from *Thellungiella salsuginea* has a potential dimerization state when it is associated with a hydrophobic surface.

To map the regions responsible for the OpsDHN1-OpsDHN1 interaction, three versions that exclude conserved regions of the OpsDHN1 protein were generated and the direct interactions were evaluated. We found that the interaction of OpsDHN1-OpsDHN1 requires residues 98–248, which contains the three K-segments and the histidine-rich motif. These data are supported by the following observations: (i) deletion of a region that includes the last K-segment in one version (SK_3_/SK_2_ or SK_2_/SK_3_) decreases OpsDHN1 dimer interaction (Figure 3); (ii) the interaction between two versions that lack the C-terminal (SK_2_/SK_2_) region was abated (Figure 3); (iii) no interaction was observed with the S construct (which does not include the three segments K and histidine-rich region) and other versions such as SK_3_, SK_2_, and S (Figure 3); (iv) deletion of the histidine-rich motif in a OpsDHN1 monomer caused a strong decrease in the DHN-DHN interaction; moreover, the deletion of this motif in both versions S(ΔH)K_3_/S(ΔH)K_3_, resulted in a loss of interaction (Figure 4).

It has been reported using *in vitro* studies that K-segments present in DHNs proteins can adopt an amphipathic class A α-helical conformations as a response to changes in temperature (Rahman et al., 2010), pH (Eriksson et al., 2011), accessibility of water (Mouillon et al., 2008) or binding to macromolecules (Koag et al., 2003, 2009). Within this amphipathic structure, hydrophobic and hydrophilic amino acids are distributed in particular sectors of the helix. This characteristic K-segments structure is associated with macromolecular interaction with membranes and lipids (Rahman et al., 2010; Eriksson et al., 2011). Two studies have reported that K-segments are important in the cryoprotection of LDH by DHNs (Reyes et al., 2008; Drira et al., 2013). In contrast, in our experimental study (Hughes et al., 2013), we compared the cryoprotective effect of a DHN consisting of the K-ϕ-K architecture with one containing only two K-segments (K-K architecture). The K-K dehydrin had poorer activity than the K-ϕ-K dehydrin, which shows that the ϕ-segment also contributes to the cryoprotective effect (Hughes et al., 2013). We also showed that the longer the natural DHN, the more effective the protection, regardless of the Y-, S-, and K-segment numbers, also suggesting that the entire protein is involved in cryoprotection. We propose that the discrepancies between our paper and others may be that the length of the deletion can explain the activity losses.

It well known that some dehydrins can bind metals through histidine residues, making these proteins crucial for maintaining metal homeostasis in response to metal stress in plants (Hara et al., 2005; Sun and Lin, 2010). Hara et al. (2013) suggested a self-associate state *in vitro* for a KS-type DHN (AtHIRD11) in presence of Cu^2+^, and stated that it did not resemble typical aggregation. It is likely that the presence Cu^2+^ promotes DHN-DHN interactions through histidine residues; additionally these residues could regulate other functions, such as a lipid interaction via the His on/off switch in a pH dependent manner (Eriksson et al., 2011) and DNA binding mediated by Zn^2+^ (Hara et al., 2009). In this sense, we suggest that histidine rich motif play a crucial role in OpsDHN1-OpsDHN1 protein interaction, as well as other conserved domains present in the region comprising amino acids 98–248.

The cloning and purification of recombinant OpsDHN1 from *E. coli* gave us the opportunity to study some of its *in vitro* properties, especially to determine whether OpsDHN1 behaves similarly to other dehydrins. The CD spectrum of OpsDHN1 in buffer alone (Figure 5A, filled circles), with its global minimum centered around 200 nm, shows that this protein is predominantly random coil and has no notable secondary structure. This is a well-known property of DHNs, and is reflective of the lack of hydrophobic residues and large number of polar and charged residues (Tompa, 2002; Uversky, 2002). However, many IDPs have been shown to gain some structure when bound to a ligand. In the case of DHNs, this has been speculated to occur through membrane binding (Close, 1997). As has been shown for maize (Koag et al., 2003) and soybean (Soulages et al., 2003), the addition of the SDS micelles as an *in vitro* membrane mimetic caused them to gain α-helicity with a concomitant loss of coil structure, which is the same thing that happens with OpsDHN1 (Figure 5A, filled squares).

We also examined the ability of OpsDHN1 to protect LDH from freeze/thaw damage (Figure 5B). The experiment here compares the effectiveness of OpsDHN1 with K_2_, a simple model dehydrin from *Vitis riparia* that we have previously used in this assay (Hughes and Graether, 2011; Hughes et al., 2013). The plot shows that the larger sized OpsDHN1 is considerably more efficient at protecting the enzyme than K_2_, a result that fits with the idea that dehydrins are able to act as molecular shields (Hughes et al., 2013). As a negative control, we used recombinant FROST protein from *D. melanogaster* (Goto, 2001). FROST is also intrinsically disordered and of approximately the same molecular weight as OpsDHN1; however, it is rather poor at protecting LDH since it only recovers ~20% of the activity even at relatively high concentrations of FROST protein. The main difference between FROST and OpsDHN1 is that FROST has many negatively charged glutamate residues. This would suggest that the positively charged K-segments of dehydrins may be important for effective protection of LDH.

Given the large number of histidine residues in OpsDHN1, and its effects on dimerization in the yeast two-hybrid assay, we examined the effect of a divalent metal (zinc) on the protein's structure. In the absence of the metal, size-exclusion chromatography shows that the protein has an apparent molecular weight of 135 kDa (Table 1). This is considerably higher than the 28.3 kDa predicted by the cloned sequence, but is not unexpected. Calibration of size-exclusion column is based on globular proteins such as BSA and lysozyme, whereas OpsDHN1 is an intrinsically disordered protein. The unfolded nature of IDPs gives them a very extended structure, such that it will migrate as a much larger protein than their actual molecular weight. Interestingly, the addition of zinc had two effects: one is that a large, broad peak was observed centered around 1000 kDa, which likely represents the aggregation of OpsDHN1. Another minor peak shows OpsDHN1 eluting at an apparent molecular weight of 65.2 kDa, suggesting that the protein is compacting. However, the addition of 1 mM zinc caused association of the protein such that no CD signal was observed (data not shown). The simultaneous addition of zinc and SDS to OpsDHN1 showed a CD spectrum similar to that of SDS alone (Figure 5B, open squares), suggesting that the gain of secondary structure may prevent this dehydrin from aggregating. Taken all together, these results suggest that zinc binding affects the structure of OpsDHN1 and its oligomeric state. We speculate that lower concentrations may cause both compaction of the protein and promote dimerization, however this requires experimental confirmation.

Two models have been proposed for DHNs to protect cellular proteins or enzymes against drying, freezing, or heat-induced aggregation: the molecular shield (Tunnacliffe and Wise, 2007; Hughes and Graether, 2011; Hughes et al., 2013) and the disordered chaperone (Tompa and Kovacs, 2010). In the molecular shield model, it has suggested that there is no direct interaction between DHNs and unfolded proteins; however, it is proposed that the cryoprotective function of DHNs may be due to the presence of DHNs in the space around unfolded proteins to form a physical barrier to avoid aggregation caused by stressful environments (Hincha and Thalhammer, 2012). In the second model, DHNs could act as a disordered chaperone, assisting in the folding and preventing the aggregation of other proteins by a variety of mechanisms, from an indirect interaction due to its high capacity to bind water, to a direct interaction with hydrophilic surfaces in the native state of proteins, or through the recognition of hydrophobic patches exposed in a partially denatured protein.

Our data on the DHN-DHN interaction as well as the disordered structure of OpsDHN1 protein suggest some insights about the molecular mechanism of how these proteins avert the adverse effects inside the cell. DHNs could function through the formation of dimers and large multimeric complexes, forming a large molecular shield around its biological targets, capable of stabilizing protein species in a partially unfolded state, thereby preventing aggregation until the stress has abated.

### Conflict of interest statement

The authors declare that the research was conducted in the absence of any commercial or financial relationships that could be construed as a potential conflict of interest.

## Supplementary material

Supplemental Figure 1 **Principle of split-ubiquitin yeast two-hybrid system**. OpsDHN1 protein was inserted into the membrane protein Ost4p and the C-terminal half of ubiquitin (Cub-bait construct), followed by the artificial transcription factor LexA-VP16. Proper insertion of bait constructs into the membrane and co-expression with NubI vector results in activation of the reporter genes (as measured by growth of white colonies on semi-selective medium (SD-LW), selective medium (SD-LWHA), and color development in a β-galactosidase assay) because wild-type NubI has a strong affinity for Cub. On the other hand, co-expression with NubG does not activate the reporter genes, since this portion bearing the isoleucine to glycine mutation at position 13 shows no affinity for Cub. However, if OpsDHN1 SK_3_-Cub/OpsDHN1SK_3_-NubG interacts, Cub and NubG complement to form split-ubiquitin, followed by cleavage and translocation of LexA-VP16 to the nucleus and transcriptional activation of endogenous reporter genes (*ADE2*, *HIS3*, and *LacZ*).

Supplemental Figure 2 **Control assay of OpsDHN1 baits constructs**. Co-transformed yeast cells with control system vectors NubI positive, NubG negative, and pPR3-N and with bait constructs (Cub): **(A)** SK_3_-Cub, **(B)** SK_2_-Cub, **(C)** S-Cub, and **(D)** S(ΔH) K_3_- Cub were spotted at an OD_600_ of 0.8 and at serial 10-fold dilutions on semi-selective (SD-LW) and on selective (SD-LWHA) media. Quantitative β-Galactosidase activity was assayed by hydrolysis of the *o*-nitrophenyl-b-galactoside (ONPG), as expressed in nmol ONP/min per mg of protein. Data represent the mean ± *SD*, (*n* = 3).

Supplemental Figure 3 **OpsDHN1 protein contains 19 histidines**. OpsDHN1 amino acid sequence, histidine residues are highlighted in yellow throughout the sequence.
