# Supplementary figures and images for: A dehydrin-dehydrin interaction: the case of SK3 from Opuntia streptacantha
Source: Front Plant Sci. 2014 Oct 10;5:520. doi: 10.3389/fpls.2014.00520 (PMC4193212; doi:10.3389/fpls.2014.00520)

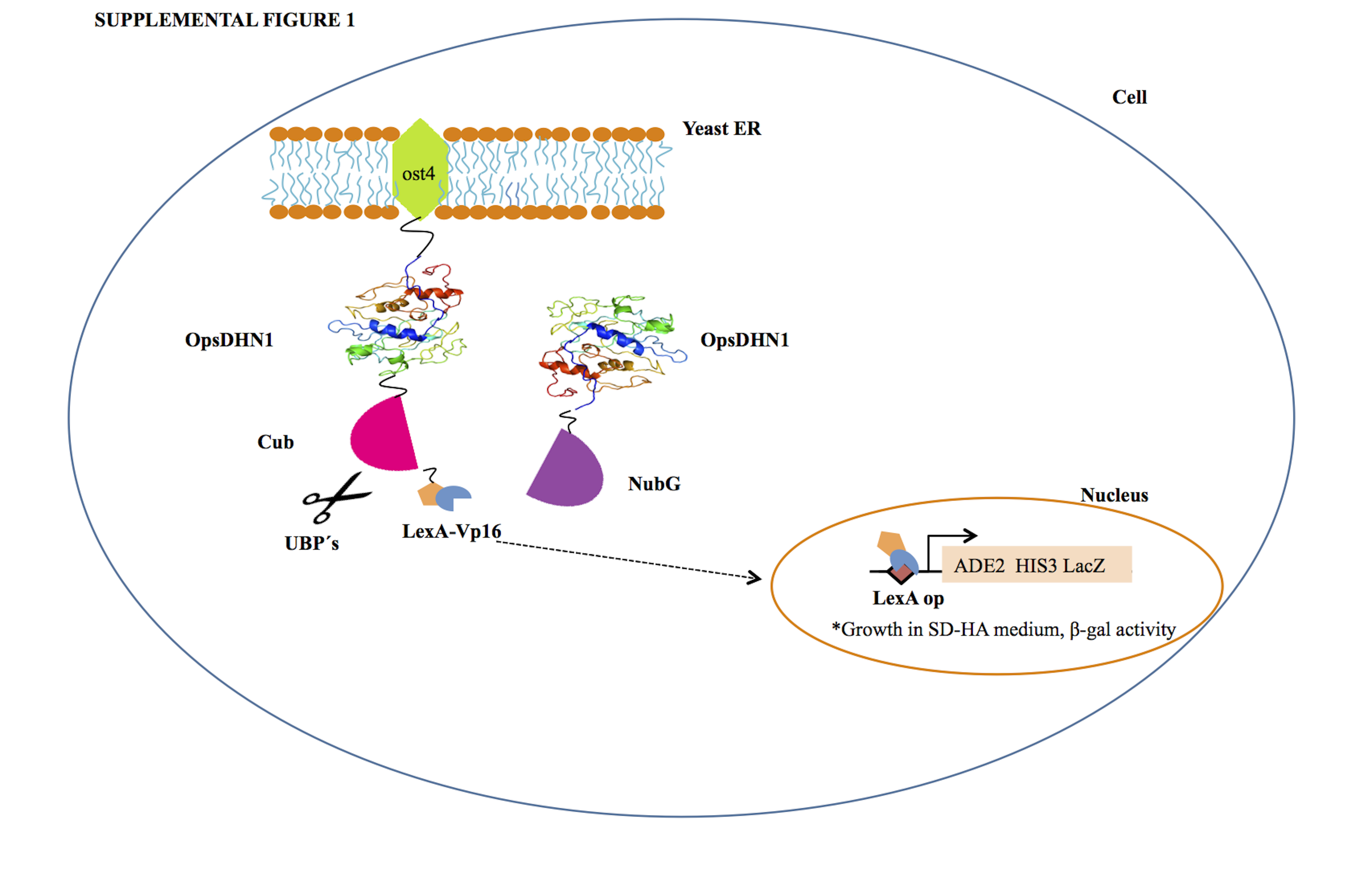

Supplement: Supplemental Figure 1 — Principle of split-ubiquitin yeast two-hybrid system. OpsDHN1 protein was inserted into the membrane protein Ost4p and the C-terminal half of ubiquitin (Cub-bait construct), followed by the artificial transcription factor LexA-VP16. Proper insertion of bait constructs into the membrane and co-expression with NubI vector results in activation of the reporter genes (as measured by growth of white colonies on semi-selective medium (SD-LW), selective medium (SD-LWHA), and color development in a β-galactosidase assay) because wild-type NubI has a strong affinity for Cub. On the other hand, co-expression with NubG does not activate the reporter genes, since this portion bearing the isoleucine to glycine mutation at position 13 shows no affinity for Cub. However, if OpsDHN1 SK3-Cub/OpsDHN1SK3-NubG interacts, Cub and NubG complement to form split-ubiquitin, followed by cleavage and translocation of LexA-VP16 to the nucleus and transcriptional activation of endogenous reporter genes (ADE2, HIS3, and LacZ). [file Image1.TIFF]

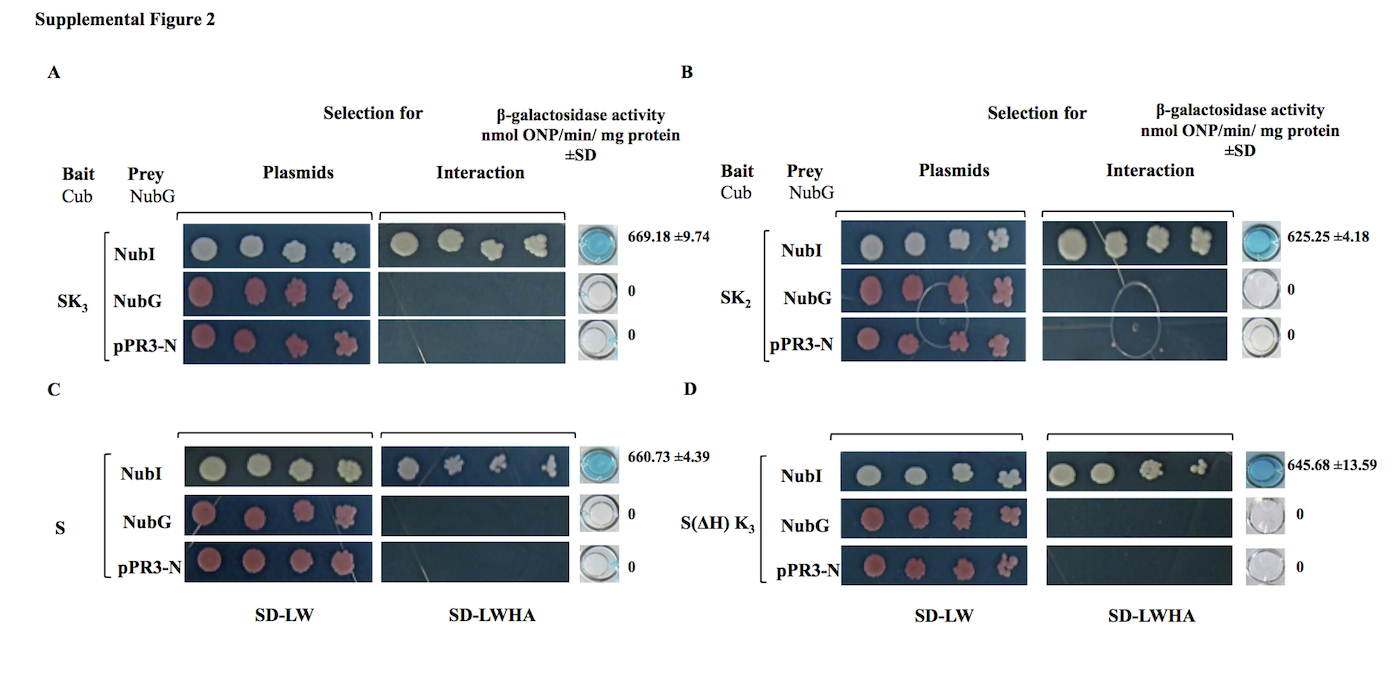

Supplement: Supplemental Figure 2 — Control assay of OpsDHN1 baits constructs. Co-transformed yeast cells with control system vectors NubI positive, NubG negative, and pPR3-N and with bait constructs (Cub): (A) SK3-Cub, (B) SK2-Cub, (C) S-Cub, and (D) S(ΔH) K3- Cub were spotted at an OD600 of 0.8 and at serial 10-fold dilutions on semi-selective (SD-LW) and on selective (SD-LWHA) media. Quantitative β-Galactosidase activity was assayed by hydrolysis of the o-nitrophenyl-b-galactoside (ONPG), as expressed in nmol ONP/min per mg of protein. Data represent the mean ± SD, (n = 3). [file Image2.TIFF]

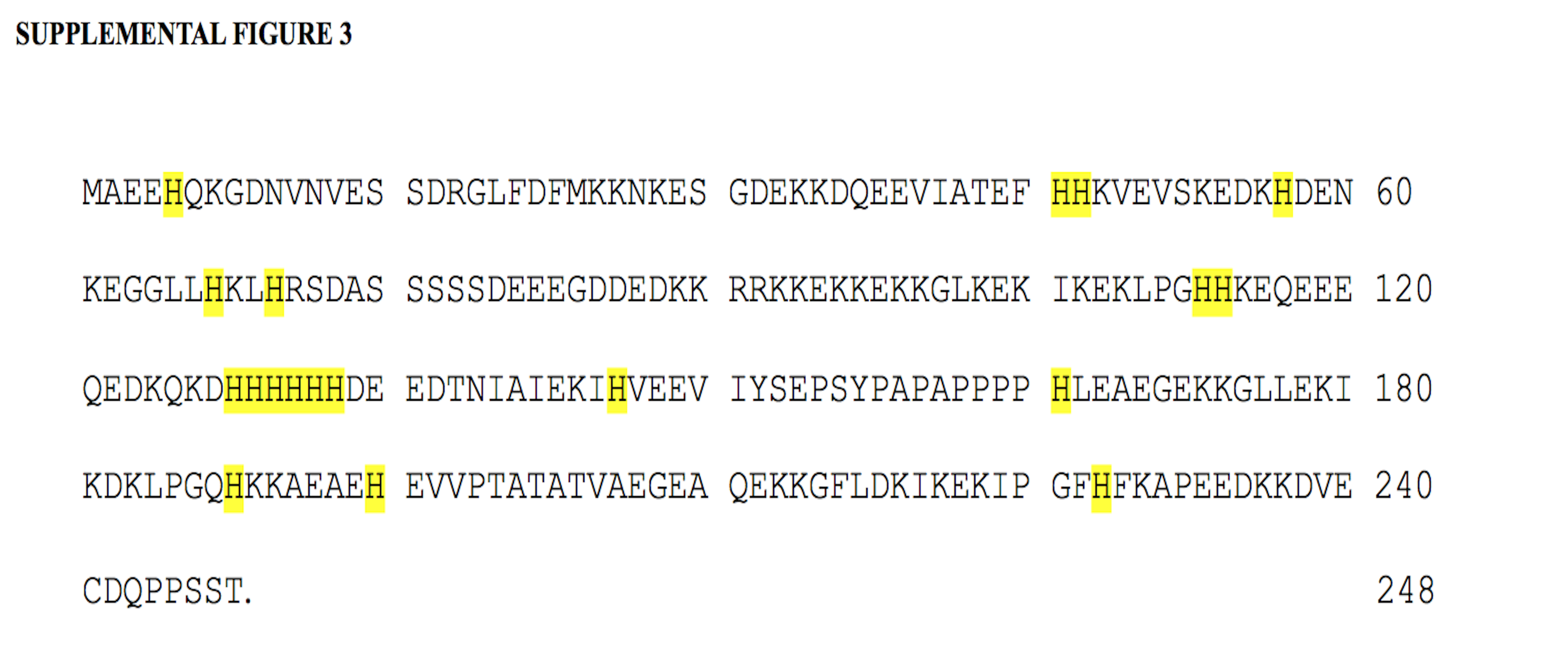

Supplement: Supplemental Figure 3 — OpsDHN1 protein contains 19 histidines. OpsDHN1 amino acid sequence, histidine residues are highlighted in yellow throughout the sequence. [file Image3.TIFF]
