# Supplementary material for: A dehydrin-dehydrin interaction: the case of SK3 from Opuntia streptacantha
Source: Front Plant Sci. 2014 Oct 10;5:520. doi: 10.3389/fpls.2014.00520 (PMC4193212; doi:10.3389/fpls.2014.00520)
Supplement: Supplementary file 4 [file Table1.PDF]

Supplemental Table 1 Generated strains for yeast two-hybrid system.

Parental strain	Construction	Auxotrophy	Strain
NMY51: MATahis3D200 trp1-901 leu2-3,112 ade2 LYS2::$(lexAop)_4$-HIS3 ura3::$(lexAop)_8$-lacZ ade2::$(lexAop)_8$-ADE2 GAL4 (Dualsystems Biotech AG)	pDHB1-OpsDHN1 SK_3-Cub<br><br>pPR3-N-OpsDHN1 SK_3-NubG	*LEU2 TRP1*	NMY51OpsDHN1 SK_3-Cub/NubG
	pDHB1-OpsDHN1 SK_3-Cub<br><br>pPR3-N-OpsDHN1 SK_2-NubG	*LEU2 TRP1*	NMY51OpsDHN1SK_3-Cub/SK_2-NubG
	pDHB1-OpsDHN1 SK_3-Cub<br><br>pPR3-N-OpsDHN1 S-NubG	*LEU2 TRP1*	NMY51OpsDHN1SK_3-Cub/S-NubG

	pDHB1-OpsDHN1 SK_2-Cub<br><br>pPR3-N-OpsDHN1 SK_3-NubG	*LEU2*<br>*TRP1*	NMY51OpsDHN1SK_2-Cub/SK_3-NubG
	pDHB1-OpsDHN1 SK_2-Cub<br><br>pPR3-N-OpsDHN1 SK_2-NubG	*LEU2*<br>*TRP1*	NMY51OpsDHN1SK_2-Cub/SK_2-NubG
	pDHB1-OpsDHN1 SK_2-Cub<br><br>pPR3-N-OpsDHN1 S-NubG	*LEU2*<br>*TRP1*	NMY51OpsDHN1SK_2-Cub/S-NubG
	pDHB1-OpsDHN1 S-Cub<br><br>PPR3-N-OpsDHN1 S-NubG	*LEU2*<br>*TRP1*	NMY51OpsDHN1S-Cub/S-NubG
	pDHB1-OpsDHN1 S(ΔH)K_3-Cub<br><br>pPR3-N-OpsDHN1 SK_3-NubG	*LEU2*<br>*TRP1*	NMY51OpsDHN1S(ΔH)K_3-Cub/SK_3-NubG
	pDHB1-OpsDHN1		

	S(ΔH)K_3-Cub<br><br>pPR3-N-OpsDHN1 S(ΔH)K_3-NubG	*LEU2*<br>*TRP1*	NMY51OpsDHN1S(ΔH)K_3-Cub/ S(ΔH)K_3-NubG
	pDHB1-OpsDHN1 S(ΔH)K_3-Cub<br><br>pPR3-N-OpsDHN1 SK_2-NubG	*LEU2*<br>*TRP1*	NMY51OpsDHN1S(ΔH)K3-Cub/ SK_2-NubG
	pDHB1-OpsDHN1 S(ΔH)K_3-Cub<br><br>pPR3-N-OpsDHN1 S-NubG	*LEU2*<br>*TRP1*	NMY51OpsDHN1S(ΔH)K_3-Cub/ S-NubG
	pDHB1-OpsDHN1 SK_3-Cub<br><br>pPR3-N-OpsDHN1 S(ΔH)K_3-Nub-G	*LEU2*<br>*TRP1*	NMY51OpsDHN1SK_3-Cub/ S(ΔH)K_3-NubG
	pDHB1-OpsDHN1 SK_2-Cub<br><br>pPR3-N-OpsDHN1 S(ΔH)K_3-Nub-G	*LEU2*<br>*TRP1*	NMY51OpsDHN1SK_2-Cub/ S(ΔH)K_3-NubG

	pDHB1-OpsDHN1 S-Cub<br><br>pPR3-N-OpsDHN1 S(ΔH)K_3-Nub-G	*LEU2*<br>*TRP1*	NMY51OpsDHN1S-Cub/S(ΔH)K_3-NubG
	pDHB1-OpsDHN1 SK_3-Cub<br><br>pAl-Alg5 (Dualsystembiotech)	*LEU2*<br>*TRP1*	NMY51OpsDHN1SK_3-Cub/NubI
	pDHB1-OpsDHN1 SK_3-Cub<br><br>pDL2-Alg5 (Dualsystembiotech)	*LEU2*<br>*TRP1*	NMY51OpsDHN1SK_3-Cub/NubG
	pDHB1-OpsDHN1 SK_3-Cub<br><br>pPR3-N (Dualsystembiotech)	*LEU2*<br>*TRP1*	NMY51OpsDHN1SK_3-Cub/pPR3-N
	pDHB1-OpsDHN1 SK_2-Cub<br><br>pAl-Alg5 (Dualsystembiotech)	*LEU2*<br>*TRP1*	NMY51OpsDHN1SK_2-Cub/NubI

	pDHB1-OpsDHN1 SK_2-Cub<br><br>pDL2-Alg5 (Dualsystembiotech)	*LEU2*<br>*TRP1*	NMY51OpsDHN1SK_2-Cub/NubG
	pDHB1-OpsDHN1 SK_2-Cub<br><br>pPR3-N (Dualsystembiotech)	*LEU2*<br>*TRP1*	NMY51OpsDHN1SK_2-Cub/pPR3-N
	pDHB1-OpsDHN1 S-Cub<br><br>pAl-Alg5 (Dualsystembiotech)	*LEU2*<br>*TRP1*	NMY51OpsDHN1S-Cub/NubI
	pDHB1-OpsDHN1 S-Cub<br><br>pDL2-Alg5 (Dualsystembiotech)	*LEU2*<br>*TRP1*	NMY51OpsDHN1S-Cub/NubG
	pDHB1-OpsDHN1 S-Cub<br><br>pPR3-N (Dualsystembiotech)	*LEU2*<br>*TRP1*	NMY51OpsDHN1S-Cub/pPR3-N
	pDHB1-OpsDHN1		

	S(ΔH)K_3-Cub<br><br>pAl-Alg5<br>(Dualsystembiotech)	*LEU2*<br>*TRP1*	NMY51OpsDHN1S(ΔH)K_3-Cub/NubI
	pDHB1-OpsDHN1<br>S(ΔH)K_3-Cub<br><br>pDL2-Alg5<br>(Dualsystembiotech)	*LEU2*<br>*TRP1*	NMY51OpsDHN1S(ΔH)K_3-Cub/NubG
	pDHB1-OpsDHN1<br>S(ΔH)K_3-Cub<br><br>pPR3-N<br>(Dualsystembiotech)	*LEU2*<br>*TRP1*	NMY51OpsDHN1S(ΔH)K_3-Cub/pPR3-N
	pDHB1-largeT<br>(Dualsystembiotech)<br><br>pDSL-Δp53<br>(Dualsystembiotech)	*LEU2*<br>*TRP1*	NMY51positive interaction control
