# Supplementary material for: A dehydrin-dehydrin interaction: the case of SK3 from Opuntia streptacantha
Source: Front Plant Sci. 2014 Oct 10;5:520. doi: 10.3389/fpls.2014.00520 (PMC4193212; doi:10.3389/fpls.2014.00520)
Supplement: Supplementary file 5 [file Table2.DOCX]

| **Plasmids** | **Construction** | **Oligonucleotides** | **Length aa** |
| --- | --- | --- | --- |
| pDHB1 | OpsDHN1-SK_3_ | 1.BOpsDHN1-SK_3_-F (5’ ATTAACAAGGCCATTACGGCCATGGCGGAAGAACACCAAAA 3’) | 248 |
|  |  | 2.BOpsDHN1-SK_3_R (5’ AACTGATTGGCCGAGGCGGCCAAAGTTGATGAAGGGGGTTGA 3’) |  |
|  |  |  |  |
| pDHB1 | OpsDHN1-SK_2_ | 1.BOpsDHN1-SK_3_-F (5’ ATTAACAAGGCCATTACGGCCATGGCGGAAGAACACCAAAA 3’) | 199 |
|  |  | 3.BOpsDHN1-SK_2_-R (5´AACTGATTGGCCGAGGCGGCCTTGGGACCACCTCGTG 3´) |  |
|  |  |  |  |
| pDHB1 | OpsDHN1-S | 1.BOpsDHN1-SK_3_-F (5´ATTAACAAGGCCATTACGGCCATGGCGGAAGAACACCAAAA 3´) |  |
|  |  | 4.BOpsDHN1-S-R (5´AACTGATTGGCCGAGGCGGCCAATTTCTTCTCCTTCTTCCTCC 3´) | 97 |
|  |  |  |  |
| pDHB1 | OpsDHN1-S(ΔH)K_3_ | 1.BOpsDHN1-SK_3_-F (5’ ATTAACAAGGCCATTACGGCCATGGCGGAAGAACACCAAAA 3’) | 223 |
|  |  | 5.OpsDHN1-∆H-R (5’ GGGGTACCTGGAAGTTTCTCCTTAATCTTC 3’) |  |
|  |  |  |  |
|  |  | 6.BOpsDHN1-∆H-F (5’-GGGGTACCAATATAGCAATAGAGAAGATCCAC-3’) |  |
|  |  | 2.BOpsDHN1-SK_3_-R (5’ AACTGATTGGCCGAGGCGGCCTTAAGTTGATGAAGGGGGTTGAT 3’) |  |
|  |  |  |  |
| pPR3-N | OpsDHN1-SK_3_ | 1.BOpsDHN1-SK_3_-F (5’ ATTAACAAGGCCATTACGGCCATGGCGGAAGAACACCAAAA 3’) | 249 |
|  |  | 7.POpsDHN1-SK_3_-R (5’ AACTGATTGGCCGAGGCGGCCTTAAGTTGATGAAGGGGGTTGAT 3’) |  |
|  |  |  |  |
| pPR3-N | OpsDHN1-SK_2_ | 1.BOpsDHN1-SK_3_-F (5’ ATTAACAAGGCCATTACGGCCATGGCGGAAGAACACCAAAA 3’)  9.POpsDHN1-SK_2_-R (5´ AACTGATT GGCCGAGGCGGCCTTATTGGGACCACCTCGT 3´) | 200 |
|  |  |  |  |
| pPR3-N | OpsDHN1-S | 1.BOpsDHN1-SK_3_-F (5’ ATTAACAAGGCCATTACGGCCATGGCGGAAGAACACCAAAA 3’) | 98 |
|  |  | 10.POpsDHN1-S-R (5´AACTGATTGGCCGAGGCGGCCTTATTTCTTCTCCTTCTTCCTCC 3´) |  |
|  |  |  |  |
| pPR3-N | OpsDHN1-S(∆H)K_3_ | 1.BOpsDHN1-SK_3_-F (5’ ATTAACAAGGCCATTACGGCCATGGCGGAAGAACACCAAAA 3’) |  |
|  |  | 5.BOpsDHN1-∆H-R (5’ GGGGTACCTGGAAGTTTCTCCTTAATCTTC 3’) | 224 |
|  |  |  |  |
|  |  | 6.BOpsDHN1-∆H-F (5’-GGGGTACCAATATAGCAATAGAGAAGATCCAC-3’) |  |
|  |  | 7.POpsDHN1-SK_3_-R (5’ AACTGATTGGCCGAGGCGGCCTTAAGTTGATGAAGGGGGTTGAT 3’) |  |
|  |  |  |  |
|  |  |  |  |

**Supplemental Table 2**
